# Supplementary material for: Schistosoma japonicum Tyrosine Hydroxylase is promising targets for immunodiagnosis and immunoprotection of Schistosomiasis japonica
Source: PLoS Negl Trop Dis. 2023 Jun 5;17(6):e0011389. doi: 10.1371/journal.pntd.0011389 (PMC10270640; doi:10.1371/journal.pntd.0011389)
Supplement: S1 Table — (DOCX) [file pntd.0011389.s001.docx]

**S1 Table.** Clinical characteristics of the enrolled subjects whose sera were used in ELISA for diagnosis.

| Subjects | Schistosomiasis  patients  n=20 | Echinococcosis  patients  n=15 | Healthy  individuals  n = 20 |
| --- | --- | --- | --- |
| Age mean* (years)  Age range (years)  Male/Female  EPG^#^ mean*  EPG^#^ range  Province  Race | 51.84±8.22  35-62  19/1  24±40.13  3-169  Hunan,  Chinese | 53.87±10.99  33-70  14/1  ——  ——  Xinjiang,  Chinese | 50.0±13.02  28-70  18/2  ——  ——  Heilongjiang,  Chinese |

* Median ± SD.

^#^ Eggs per gram of stool (assessed by Kato-katz technique)
