# Supplementary figures and images for: Schistosoma japonicum Tyrosine Hydroxylase is promising targets for immunodiagnosis and immunoprotection of Schistosomiasis japonica
Source: PLoS Negl Trop Dis. 2023 Jun 5;17(6):e0011389. doi: 10.1371/journal.pntd.0011389 (PMC10270640; doi:10.1371/journal.pntd.0011389)

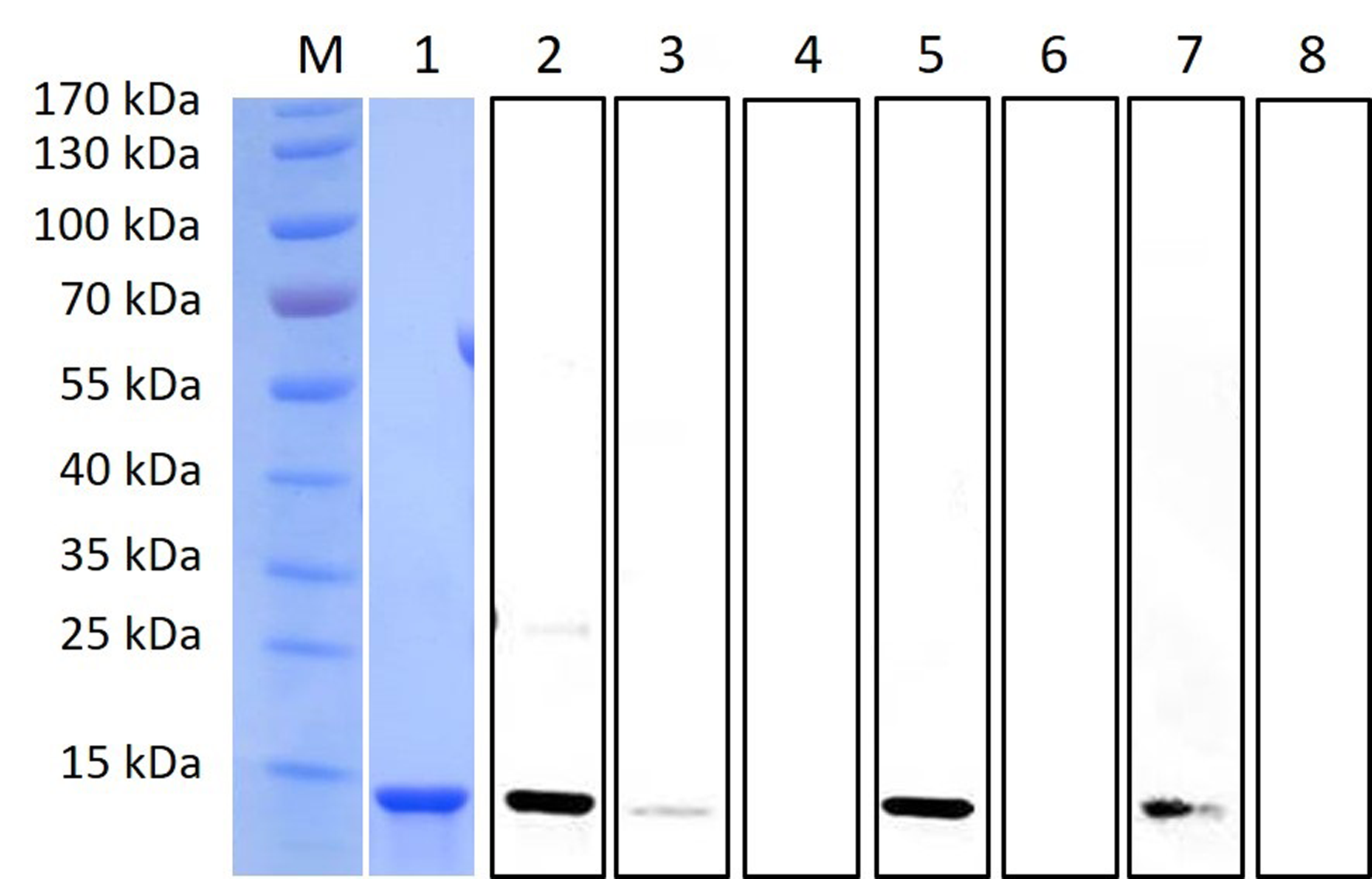

Supplement: S1 Fig — Recombinant protein of SjTH (71–181 amino acids) were resolved in 12% SDS-PAGE and stained by Coomassie brilliant blue staining (Lane 1), and then detected by western blot with an anti-His tag mouse monoclonal antibody (Lane 2), a mixture of serum samples (equal volumes) from 10 schistosomiasis japonica patients (Lane 3) or 10 healthy volunteers (lane 4), a mixture of serum samples (equal volumes) from 6 infected BALB/c mice 42 days p.i. (Lane 5) or normal mice (Lane 6), and a mixture of serum samples (equal volumes) from 5 infected rabbits 42 days p.i. (Lane 7) or normal rabbits (Lane 8). (TIF) [file pntd.0011389.s002.tif]

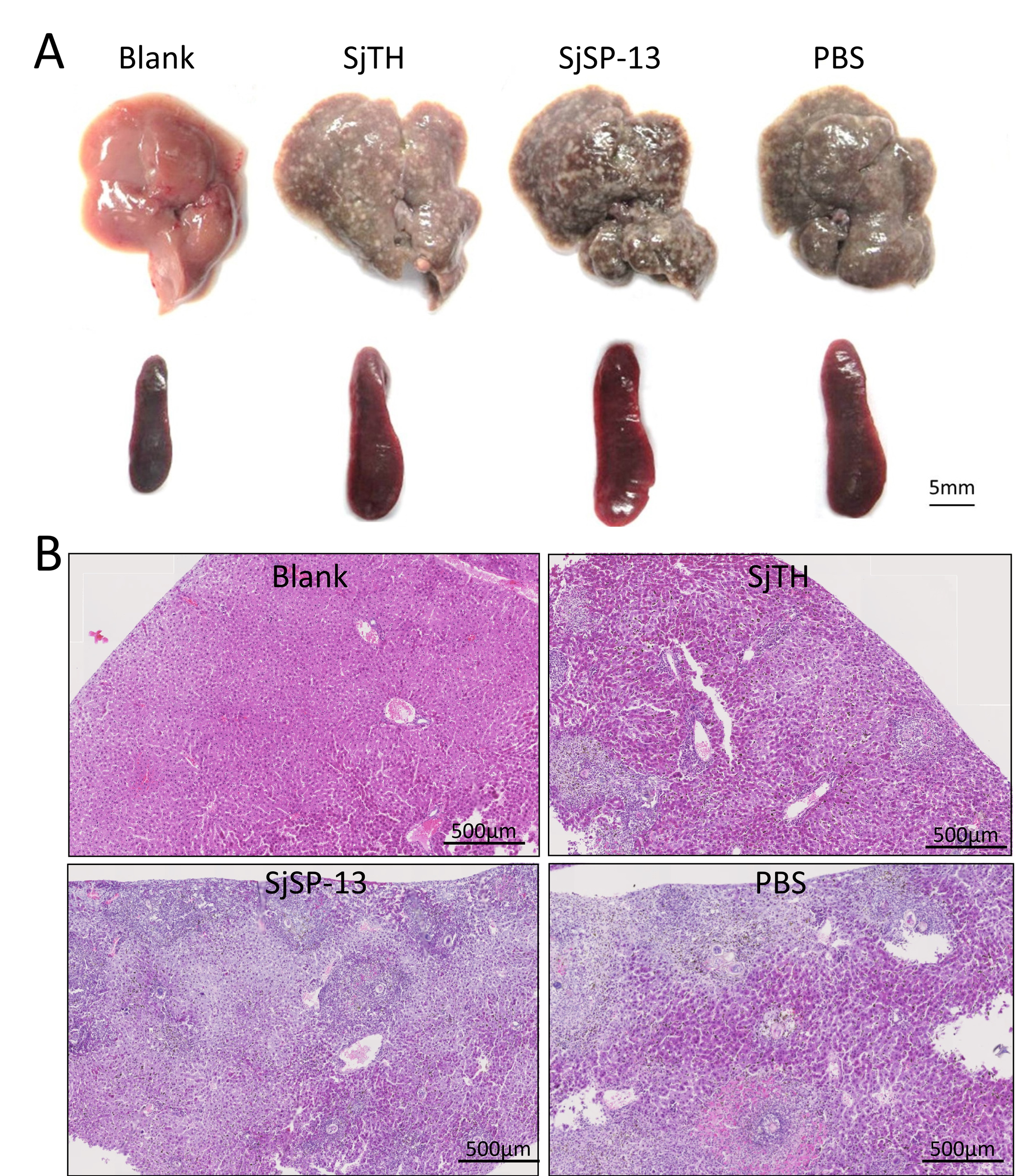

Supplement: S2 Fig — Mice (n = 10 per group) were immunized with His-tagged recombinant SjTH, SjSP-13, or PBS as control, and were then challenged with cercariae (40 ± 2 per mouse). The blank group was immunized with PBS and received no cercariae. Results are representative of two independent experiments. (A) The morphology of the livers and spleen from each group of mice are shown. (B) Granuloma formation was detected by haematoxylin-eosin staining. (TIF) [file pntd.0011389.s003.tif]
