# Supplementary material for: Schistosoma japonicum Tyrosine Hydroxylase is promising targets for immunodiagnosis and immunoprotection of Schistosomiasis japonica
Source: PLoS Negl Trop Dis. 2023 Jun 5;17(6):e0011389. doi: 10.1371/journal.pntd.0011389 (PMC10270640; doi:10.1371/journal.pntd.0011389)
Supplement: Attachment — Submitted filename: Review Report.docx [file pntd.0011389.s004.docx]

**Review Report**

**Manuscript Title: Schistosoma japonicum Tyrosine Hydroxylase is Promising Targets for Immunodiagnosis and Immunoprotection of Schistosomiasis japonica**

1. This research is cited as novel research since there is limited information pertaining to SjTH and the potential role in immunodiagnosis and immunoprotection in the context of invivo studies.
2. The discussion and results include a narrative on the scarceness of literature on THs in *S japonicum*.
3. The authors need to consider strengthening their statement in lines 311 and 312 about the “strong evidence”… supporting vaccine development and how

this relates to the present findings.

4. The introduction should contain a statement on the burden of disease related specifically for *S.japonicum.*
5. On line 319, and 344 please correct to *S.japonicum.*
6. **Fig 1. Expression of SjTH in parasites at different developmental stages-** line 546- should the narrative not also include the “s” group
7. S1 Table: could the patients egg counts not be included?
8. Please can the authors mention any limitations of the study.
9. Can the authors include a few more recent references if possible on the main subject matter.
10. The authors are advised to consult the review paper below which covers the narratives on the advances in diagnostic research in S.japonicum, this may strengthen their background into what has been done regarding this parasite.

“Cheng Chen, Qinghong Guo, Zhiqiang Fu, Jinming Liu, Jiaojiao Lin, Kai Xiao, Pengxiang Sun, Xiaonan Cong, Runxia Liu, Yang Hong,

Reviews and advances in diagnostic research on Schistosoma japonicum,

Acta Tropica,

Volume 213,

2021,

105743,

ISSN 0001-706X,

https://doi.org/10.1016/j.actatropica.2020.105743.

(https://www.sciencedirect.com/science/article/pii/S0001706X20316569)

Abstract: Schistosomiasis is an acute and chronic parasitic disease caused by blood flukes (trematode worms) of the genus Schistosoma. Schistosoma japonicum (S. japonicum) infection has decreased significantly in prevalence and intensity of infection in China. However, this disease still remains a serious public health problem in some endemic areas of the Philippines and Indonesia. Thus, more accurate and sensitive methods are much needed for further control of this disease. Here, we review the research progress in techniques for the diagnosis of S. japonicum infection.

Keywords: Schistosomiasis; Schistosoma japonicum; Diagnosis; New techniques”

11. The following paper has been published in another journal- how do the findings from this paper differ from the present paper. It seems like some of the authors are common from both papers

T Journal Article

A1 Hou, Nan

A1 Piao, Xianyu

A1 Jiang, Ning

A1 Liu, Shuai

A1 Cai, Pengfei

A1 Liu, Bing

A1 McManus, Donald P

A1 Chen, Qijun

T1 Novel Hepatic Schistosomula Antigens as Promising Targets for Immunodiagnosis and Immunoprotection of Schistosomiasis japonica

JF The Journal of Infectious Diseases

JO J Infect Dis

YR 2022

DO 10.1093/infdis/jiac077

VO 225

IS 11

SP 1991

OP 2001

SN 0022-1899

AB Antigens of migrating schistosomula are promising candidates as schistosomiasis vaccine targets, since immune attack on hepatic schistosomula would interrupt the parasites life cycle and reduce egg burden on the host.In this study, we report a collection of Schistosoma japonicum schistosomula proteins (SjScPs) that are highly expressed in hepatic schistosomula. The expression characteristics, antigenicity and immune protection of these proteins were studied by western blot, ELISA, immunofluorescence and challenge assays.We found that several of these SjScPs were highly antigenic and could effectively stimulate humoral immune responses in both human and other mammalian hosts. In particular, SjScP25, SjScP37, SjScP41, SjScP80, and SjScP88 showed high potential as biomarkers for schistosomiasis immunodiagnosis. Furthermore, we demonstrated that immunization with several of the recombinant SjScPs were able to protect mice from S japonicum challenge infection, with SjScP25 generating the most protective results.Our work represents a group of novel schistosome immunogens, which may be promising schistosomiasis japonica diagnosis and vaccine candidates.

RD 1/26/2023

UL https://doi.org/10.1093/infdis/jiac077
