# Supplementary material for: Schistosoma japonicum Tyrosine Hydroxylase is promising targets for immunodiagnosis and immunoprotection of Schistosomiasis japonica
Source: PLoS Negl Trop Dis. 2023 Jun 5;17(6):e0011389. doi: 10.1371/journal.pntd.0011389 (PMC10270640; doi:10.1371/journal.pntd.0011389)
Supplement: Attachment — Submitted filename: Responses to the review comments.docx [file pntd.0011389.s005.docx]

**Point-to-point response to the reviewers' comments:**

**Reviewer #1:** (No Response)

**Reviewer #2:**

**Comment 1:** Questionable TH protein expression pattern. Previous work that are cited in the manuscript describe neuronal enrichment of catecholamines. For instance, Gianutsos et al., 1977 demonstrates that the highest enrichment of dopamine and norepinephrine is seen in the anterior region where the cephalic ganglia (central nervous system) are located. This is further supported by the fact Smp_007690 (*S. mansoni* ortholog of SjTH) mRNA is enriched in a subset of neurons in adult worms (Wendt et al., 2021). Together with reported effects of catecholamines in circular/longitudinal muscle contraction (Pax et al., 1984), these data support a model in which TH enzyme in the CNS produces catecholamines that leads to proper functioning of peripheral neuromuscular function. Thus, the antibody staining results that show protein expression in the gut lumen is questionable. Expression pattern in the CNS should be shown as a comparison, and if there is no enrichment seen in the CNS, the authors need to explain the discrepancy between the predicted and observed expression patterns. If mRNA in situ hybridization cannot be done, alternatively, the authors can show the PCA plot of the single cell RNA-seq atlas available for *S. mansoni* (see examples shown in Hulme et al., 2022 Supplementary Figures) and discuss the biological/technical reasons for the discrepancy.

**Response:** We appreciate very much for the comment and suggestion. Our study really neglected to examine the head of the parasites, where the CNS is located. This time, we used frozen sections of schistosome head for immunofluorescence detection and found that TH indeed expressed in the head. The results were added in the revised manuscript. Although TH most commonly expressed in the CNS, many studies have reported its presence in the peripheral nervous system as well. It was detected in gut (Olsson C., 2016), retina (Jeon JY. et al., 2019), sympathetic nervous system and adrenal medulla (Haycock JW. et al., 1985). Nerve fibers of the peripheral nervous system spread throughout the body of schistosome worm and extend to the surface (EI-Sakkary N. et al., 2018), and TH may be distributed along nerve fibers of the peripheral nervous system. Anyway, this work is a preliminary study, and the location and function of TH in Schistosoma require further research to understand the potential importance of this enzyme. We are using stereo-seq to achieve spatial transcriptomics of *S. japonicum*, and our future work may further reveal the role of TH in *S. japonicum* growth and development.

**Comment 2:** Lack of mechanistic details. Although the study aims to understand the “in vivo” role of TH, and the apparent moderate protective role shown, the authors come short on investigating the reasons behind the reduced parasite burden. Are parasites unable to migrate from the skin to the bloodstream? Are parasites’ feeding, reproductive, or locomotive behavior affected? Harvesting parasites at different times after infection and comparing the recovery efficiency would provide some answers. In parallel, monitoring the behavior of harvested parasites would give some clues. Another important mechanistic detail is that if the antibody against rSjTH indeed targets and neutralizes SjTH, one might expect to see reduced levels of catecholamine. These should nicely corroborate and support reduced parasite/egg burden results.
**Response:** We appreciate very much for your wonderful advice. We measured catecholamine levels in the worms 42 days post infection, but there was no significant difference between parasites from anti-SjTH antibody and IgG group. Schistosomula that are severely affected by anti-SjTH antibodies may be eliminated by the host immune system, resulting in fewer worms and eggs in the challenge experiments, while the ones mildly affected may successfully resist the host immune system and eventually mature, with compensatory production of catecholamine. We will test catecholamine levels in parasites at different developmental stages in our future work. Furthermore, we used RNAi technology to construct SjTH knockdown strain to reveal the detailed mechanism of SjTH, but the knockdown effect was limited. We have designed new dsRNAs and further investigation will be carried out to reveal the detailed mechanism of SjTH. Intermediate locations such as the skin or circulation are very critical stages, and the feeding, reproductive, or locomotive behavior of the parasites are essential factors in parasite viability. In our future work, we will take the recommendation and further design time ladder experiments to reveal the dynamic changes of the parasites.

**Comment 3**: Information regarding the Gene ID should be specified in the Methods: Sjp_0053450.

**Response:** Information regarding the Gene ID “*SjTH* (GenBank ID: HQ234745.1)” has been added in line 150 of the revised manuscript (highlighted version).

**Comment 4:** Figure 1 has a discrepancy in expression between SjTH mRNA and protein (especially in eggs where mRNA is highly expressed but the protein is not detected). The authors need to explain why there is such a big discrepancy in the text.

**Response:** In our previous work, we designed and prepared the whole genome expression profile chip of *S. japonicum*. Each group was repeated three times with three batches of samples. In the process of verifying the chip results by QPCR and WB, we found that the transcription and protein levels of many schistosome genes were not consistent in their expression levels, especially those from eggs. This is a very interesting phenomenon that needs to be explored further.

**Comment 5:** Figure 1. Why is y-axis of qPCR data shown in 0.001 units? Please describe step-by-step analysis on how the values were derived in the Methods section or in the Figure Legend.

**Response:** We are sorry for the unclearness. The gene expression values of the parasite at specific developmental stages were normalized to the standard control (GAPDH in the standard parasite cDNA). The Methods Section in the revised manuscript has been modified for detailed analysis.

**Comment 6:** In Figure 1, ‘hepatic schistosomula’ stage has not been defined. Indicate how many days post-infection the worms were harvested for the reported experiments.

**Response:** The hepatic schistosomula were harvested 42 days post-infection. The legend of Figure 1 has been modified to refine the information about hepatic schistosomula.

**Comment 7:** Figure 2. The image qualities are poor to really see the signal. In fact, all figures are severely pixelated.

**Response:** The immunofluorescence of worms is usually not as clear as that of animal tissues or cells. We will further optimize the experimental conditions in our future work to obtain clearer images.

**Comment 8:** Figure 2. How many worms were analyzed for each stage, and how many of those showed such expression patterns need to be indicated.

**Response:** Three batches of parasites, and at least 3 worms of each batch were analyzed for each stage, all the worms showed similar expression patterns. The legend of Figure 2 has been modified in the revised manuscript.

**Comment 9:** Figure 2. In panels in D, DAPI and IgG panels appear to be swapped.

**Response:** We are sorry for the confusion. Figure 2 has been modified to correct the panels.

**Comment 10:** Figure 2. The scale bar units should be in µm (micrometer), not µM (micromolar). **Response:** Thank you for your correction, the mistakes have been corrected.

**Comment 11:** Why was His-rSjTH used for immunization instead of epitope-cleaved rSjTH? Since His tag was not removed, in this case, a better negative control would be an epitope-only immunization.

**Response:** We appreciate very much for the comment and suggestion. The immunogenicity of His tag is low and its molecular weight is small, thus his tag generally will not affect the structure and immunogenicity of recombinant proteins, so we generally use his-tagged recombinant protein for direct immunization. We will use epitope-cleaved rSjTH in our future work to compare the differences.

**Comment 12:** Figure 4. Representative liver (highlighting the reduced pathology of granulomatous liver) and spleen images will nicely complement Figures 4B and 4C.

**Response:** We appreciate very much for the comment and suggestion. The images of livers and spleens and HE staining of liver sections have been added in S1 Figure. The comparation of granulomas areas between the groups has been added in Figure 4F.

**Comment 13:** Figure 4. The authors should add a comment regarding the sex of schistosomes infected/recovered. From how I understood the paper, the authors used mixed sex cercariae derived from infected Oncomelania. Given the relatively small number (~40) of cercariae given to each mouse, a small variation in male:female ratio could shift the egg output and/or Th1/Th2 response. This needs to be discussed in the text.

**Response:** The question you raised is critical to the construction of schistosomiasis mice model. We are sorry for the unclear description of the method. In fact, the sex of cercariae released by snails were determined using duplex real-time PCR method, and mice were percutaneously infected with 20 ± 1 male cercariae and 20 ± 1 female cercaria. This method can effectively minimize the variation in male:female ratio. The Method section has been modified in the revised manuscript.

**Comment 14:** Figure 5. The conclusion that SjTH may modulate Th1/Th2 response is overreaching. Given that rSjTH immunization results in a moderate reduction of parasite burden (shown in Figure 4), the overall reduction in immune response could simply be due to the lower number of parasites and eggs in rSjTH infected mice. This needs to be clarified in the text.

**Response:** We appreciate very much for the comment and suggestion. The description about the Th1/Th2 response in rSjTH infected mice has been modified in the Discussion Section.

**Comment 15:** Many references are missing full citation, and are not properly formatted (e.g., italicized species name).
**Response:** We appreciate very much for your careful reading and checking. The mistakes have been corrected in the revised manuscript.

**Reviewer #3:**

**Comment 1:** This research is cited as novel research since there is limited information pertaining to SjTH and the potential role in immunodiagnosis and immunoprotection in the context of in vivo studies.

**Response:** We appreciate very much for the comment and suggestion. The research purpose of this study (line 29, line 55-56, line 107-109 in highlighted version of revised manuscript) has been modified to conform to its content.

**Comment 2:** The discussion and results include a narrative on the scarceness of literature on THs in *S. japonicum*.

**Response:** So far, there are few studies on schistosome TH, and only one study on TH in *S. japonicum* has been reported. The description about SjTH has been refined in the Discussion and Results Sections of the revised manuscript (highlighted version, line 105 and 330-331).

**Comment 3:** The authors need to consider strengthening their statement in lines 311 and 312 about the “strong evidence”… supporting vaccine development and how this relates to the present findings.

**Response:** We appreciate very much for the comment and suggestion. “strong evidence” is not appropriate here, the presentation has been revised as “Evidence from clinical and preclinical studies offers hope for developing an effective vaccine for long-term protection against schistosomiasis”.

**Comment 4:** The introduction should contain a statement on the burden of disease related specifically for *S.japonicum*.

**Response:** Information about the disease burden related for *S. japonicum* was added in the first paragraph of Introduction Section.

**Comment 5:** On line 319, and 344 please correct to *S.japonicum.*

**Response:** We are here to describe the disease caused by *S. japonicum*, so we use the term “schistosomiasis japonica”.

**Comment 6:** Fig 1. Expression of SjTH in parasites at different developmental stages- line 546- should the narrative not also include the “s” group.

**Response:** Thank you for your correction. The mistake has been corrected in the revised manuscript.

**Comment 7:** S1 Table: could the patients egg counts not be included?

**Response:** The information about egg counts of patients was added in S1 table.

**Comment 8:** Please can the authors mention any limitations of the study.

**Response**: This study only provides the potential value of SjTH as an immune diagnostic and protective target for schistosomiasis, but the research on SjTH is still in the preliminary stage, the exact location of SjTH and its specific mechanism in schistosome development need to be further investigated. The text has been added in the last paragraph of the Discussion Section of the revised manuscript.

**Comment 9:** Can the authors include a few more recent references if possible on the main subject matter.
**Response:** We appreciate very much for the comment and suggestion. Recent references have been added and old references have been replaced with recent ones, such as line 69, 85, 89, 327, 330 and 401-406, in the revised manuscript (highlighted).

**Comment 10:** The authors are advised to consult the review paper below which covers the narratives on the advances in diagnostic research in *S.japonicum*, this may strengthen their background into what has been done regarding this parasite.

**Response:** We appreciate very much for the comment and suggestion. We read the article carefully, and revised the narratives on schistosomiasis diagnostic research accordingly in the Discussion Section (line 399-407 in the highlighted revised manuscript).

**Comment 11:** The following paper has been published in another journal- how do the findings from this paper differ from the present paper. It seems like some of the authors are common from both papers.

**Response:** Our team is working on schistosomiasis japonica diagnosis and vaccine targets. In our previous work, we designed and prepared the whole genome expression profile chip of *S. japonicum*. Based on the detection results of the chip, we studied the high expression genes of parasites at different developmental stages, specific genes of *S. japonicum*, enzyme genes of schistosome and so on. The paper (Nan H. et al., 2022) reports our previous work about a collection of *S. japonicum* schistosomula proteins (SjScPs) that are highly expressed in hepatic schistosomula. The present study reports a schistosome enzyme SjTH. Both papers investigated the diagnostic and immunoprotective value of different schistosome proteins.
