# Supplementary material for: Schistosoma japonicum Tyrosine Hydroxylase is promising targets for immunodiagnosis and immunoprotection of Schistosomiasis japonica
Source: PLoS Negl Trop Dis. 2023 Jun 5;17(6):e0011389. doi: 10.1371/journal.pntd.0011389 (PMC10270640; doi:10.1371/journal.pntd.0011389)
Supplement: Attachment — Submitted filename: Comments.docx [file pntd.0011389.s006.docx]

The current work titled “*Schistosoma* *japonicum* tyrosine hydroxylase is promising targets for immunodiagnosis and immunoprotection of Schistosomiasis japonica” demonstrates the role of Tyrosine hydroxylase during *Schistosoma* *japonicum* infection. A recombinant protein SjTH was produced and the corresponding polyclonal antibody was generated. The results revealed the importance of this enzyme and its distribution in the schistosomulae and adult worms, its value in immunodiagnosis and immunization.

- The article is well written, the content fulfill the aim and the discussion is logic.

- Parasites derived-EVs have the ability to manipulate the immune system, and carry many proteins that have been used as a vaccine candidate. Thus, the authors have to discuss the results of using these vesicles as vaccine at line: 355. (PMID: **34793443,** PMID: **34644290)**
